# Immunization with *Leishmania tarentolae*-derived norovirus virus-like particles elicits high humoral response and stimulates the production of neutralizing antibodies

**DOI:** 10.1186/s12934-021-01677-1

**Published:** 2021-09-24

**Authors:** Mirosława Panasiuk, Karolina Zimmer, Anna Czarnota, Katarzyna Grzyb, Magdalena Narajczyk, Grażyna Peszyńska-Sularz, Sabina Żołędowska, Dawid Nidzworski, Lilit Hovhannisyan, Beata Gromadzka

**Affiliations:** 1 Intercollegiate Faculty of Biotechnology, University of Gdańsk and Medical University of Gdańsk, Abrahama 58, 80-307 Gdańsk, Poland; 2 Laboratory of Electron Microscopy, Faculty of Biology, University of Gdańsk, Wita Stwosza 59, 80-308 Gdańsk, Poland; 3 Nano Expo Sp. z o. o., Kładki 24, 80-822 Gdańsk, Poland; 4 Tri-City Central Animal Laboratory Research and Service Center, Medical University of Gdańsk, Dębinki 1, 80-211 Gdańsk, Poland; 5 Department of in Vitro Studies, Institute of Biotechnology and Molecular Medicine, Kampinoska 25, 80-180 Gdańsk, Poland; 6 Institute of Biotechnology and Molecular Medicine, Gdańsk, Poland

**Keywords:** Norovirus, Virus-like particles, *Leishmania tarentolae*, Immune response

## Abstract

**Background:**

Noroviruses are a major cause of epidemic and sporadic acute non-bacterial gastroenteritis worldwide. Unfortunately, the development of an effective norovirus vaccine has proven difficult and no prophylactic vaccine is currently available. Further research on norovirus vaccine development should be considered an absolute priority and novel vaccine candidates are needed. One of the recent approaches in safe vaccine development is the use of virus-like particles (VLPs). VLP-based vaccines show great immunogenic potential as they mimic the morphology and structure of viral particles without the presence of the virus genome.

**Results:**

This study is the first report showing successful production of norovirus VLPs in the protozoan *Leishmania tarentolae* (*L. tarentolae*) expression system. Protozoan derived vaccine candidate is highly immunogenic and able to not only induce a strong immune response (antibody titer reached 10^4^) but also stimulate the production of neutralizing antibodies confirmed by receptor blocking assay. Antibody titers able to reduce VLP binding to the receptor by > 50% (BT_50_) were observed for 1:5–1:320 serum dilutions.

**Conclusions:**

Norovirus VLPs produced in *L. tarentolae* could be relevant for the development of the norovirus vaccine.

## Background

Noroviruses (NoVs) are positive-sense, single-stranded RNA viruses causing epidemic and sporadic cases of acute gastroenteritis globally [1, 2]. The NoVs are highly contagious agents; they can be transmitted via the fecal–oral route and often cause outbreaks in closed communities either through close contact with infected people or through consumption of contaminated food or water [3]. The current evidence is that the disease burden of NoV is high, second only to rotavirus, as a cause of severe acute gastroenteritis and diarrhea-associated mortality worldwide. NoV is estimated to cause approximately 685 million cases of acute gastroenteritis worldwide and is responsible for more than 200,000 deaths annually. The disease occurs across the age range in all settings, but incidence is the highest in young children. More than 200 million cases annually are observed in children under 5 years old worldwide, leading to over 50,000 child deaths every year, mostly in developing countries. However, NoV infections are a problem in both developing and industrialized countries causing economic losses of over 60 billion dollars worldwide due to healthcare costs and lost productivity [4].

NoVs belong to the family of *Caliciviridae* and can be classified into ten genogroups (GI-GX) which are further subdivided into 48 genotypes. Norovirus is a non-enveloped virus of T = 3 icosahedral capsid composed mainly of multiple copies of VP1 capsid protein. Despite having a very high genetic diversity most NoV infections are caused by Genogroup II, genotype 4 (GII.4) strains. GII.4 variants are associated with 70–80% of all the reported outbreaks such as Farmington_Hills_2002, GII.4 Hunter_2004, GII.4 Den Haag_2006b, GII.4 New Orleans_2009 and GII.4 Sydney_2012 [5, 6]. Between 2002 and 2012, new GII.4 viruses emerged about every 2 to 4 years, but since 2012 the same virus (GII.4 Sydney) has been the dominant strain worldwide said to be a pandemic [7]. Without an available prophylactic vaccine, NoV pandemics spread rapidly across the globe, causing great economic burdens due to medical and social expenses. Considering the substantial disease burden and the difficulty in controlling norovirus, vaccines may be an attractive and perhaps the only way to effectively control NoV in the wider community.

In 2016 the World Health Organization stated that the development of a NoV vaccine should be considered an absolute priority. Vaccine development poses huge scientific challenges and requires a large investment of funding and time. Current trends in vaccine development focus on vaccine safety and low cost of production such as VLP-based vaccines (hepatitis B virus (HBV), human papillomavirus (HPV). VLPs are morphologically and antigenically indistinguishable to native viruses but lack genetic material which makes them non-replicating. VLPs can be produced in different, easily scalable expression systems such as bacteria, yeasts, plants, insect or mammalian cells. Additionally, VLP-based vaccines induce a robust immune response that is highly similar to that elicited by a natural viral infection [8]. Currently few VLP-based vaccines are approved and available in Europe and in the USA. These include vaccines against hepatitis B: Recombivax HB® and Engerix®, and HPV vaccine: Gardasil®.

Currently, a number of NoV vaccines are being developed with only few under clinical testing. All of these products are based on the production of non-replicating VLPs or P particle subunit that shares similar surface antigenic structures to NoV in various expression systems [9]. There are only candidate vaccines with human efficacy data to date being developed [10].

In this study, we present the potential vaccine candidate based on capsid protein of pandemic strain of NoV produced in the unconventional *Leishmania tarentolae* (*L. tarentolae*) (LEXSY) expression system. This system is easy to handle, fully eukaryotic and characterized by mammalian-like protein folding and post-translational modification machinery (mammalian type N-glycosylation pattern) [11, 12]. The main advantages of the system include inexpensive growth conditions, fast growth rate and ease of handling as it is non-pathogenic for humans. Additionally, *L. tarentolae* culture can be easily scaled up and grown in bio fermenters; in effect, the recombinant protein production yield can reach several milligrams per liter of culture [11, 13]. Recent reports also confirmed a great potential of *L. tarentolae* to be employed as a live factory producing antigens inside the body of BALB/c mice. Injection of recombinant *L. tarentolae* to mice increases antigen presentation and T-cell immune responses by effectively targeting the dendritic cells and lymphoid organs [14–17].

This report shows that a low concentration of *L. tarentolae*-derived NoV VLPs induce a high humoral immune response in vaccinated mice and stimulates the production of neutralizing antibodies.

## Results

### Expression and characterization of *L. tarentolae*-derived NoV capsid protein

**Fig. 1:**
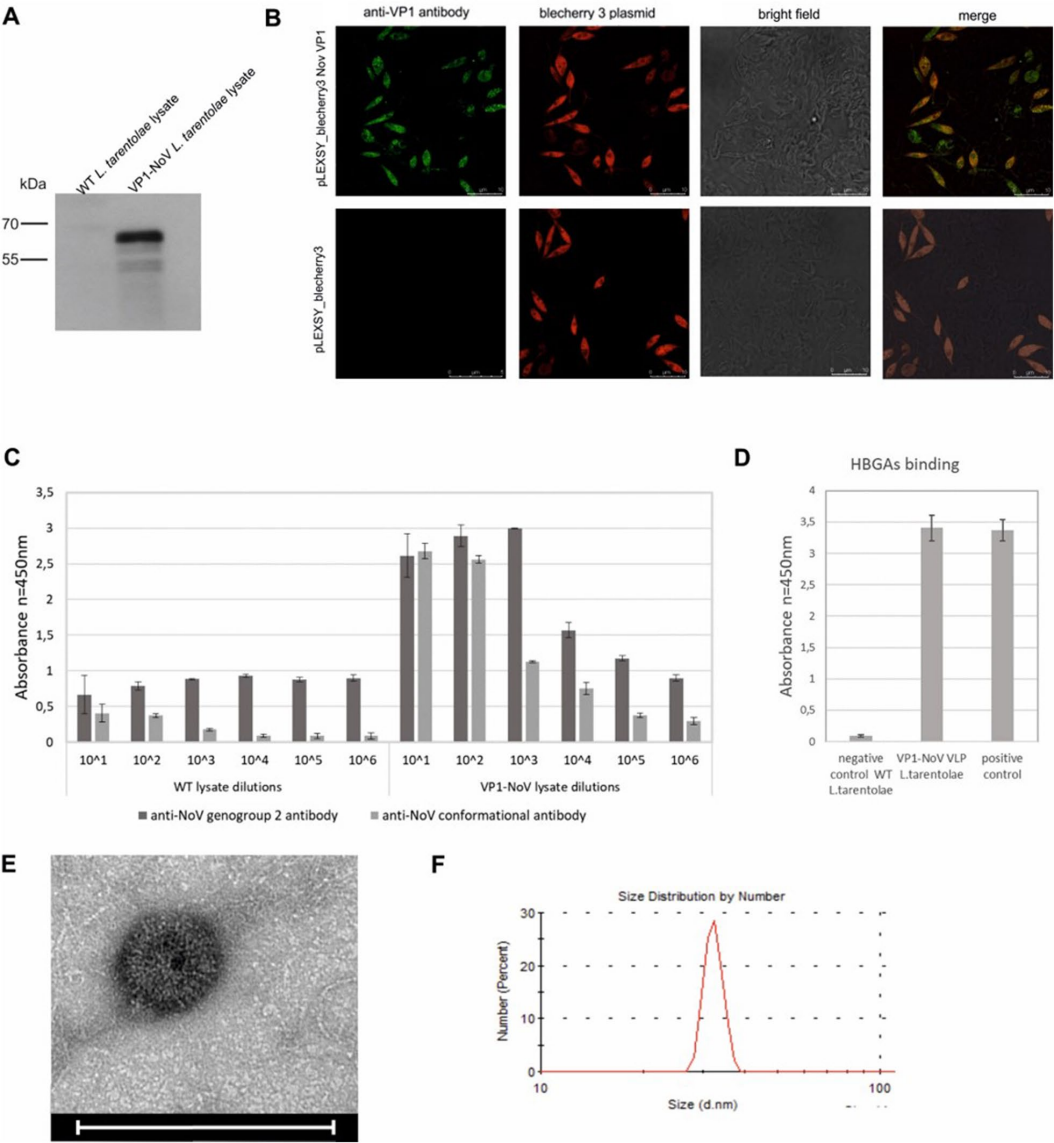
Characterization of the *L. tarentolae*-derived NoV capsid protein. **A** Western blotting analysis of the NoV VP1 expressed in the LEXSY expression system in reducing conditions. The protein was detected in cell lysates using specific anti-VP1-NoV antibodies. WT *L. tarentolae* cell lysates served as a negative control. **B** Indirect immunofluorescence of *L. tarentolae* cells expressing VP1 protein. Cells transfected with an empty pLEXSY_blecherry3 plasmid were used as a negative control. The VP1 protein was detected using anti-VP1-NoV antibodies (green). The red color corresponds to the bleachery fluorescence (transfection control). The bright-field image shows the cell morphology. Images were obtained using a Leica TCS Sp8 X confocal microscope. **C** Recognition of the NoV VP1 protein from the whole-cell lysate by ELISA. An ELISA plate was coated with serial dilutions of *L. tarentolae* cell lysates containing the VP1 protein detected with anti-NoV antibodies. WT *L. tarentolae* cell lysates served as a negative control. The bars represent the mean values obtained from triplicate experiments. **D** Binding of the NoV VP1 protein produced in *L. tarentolae* cells to HBGAs receptors. The NoV VP1 protein produced in *S. frugiperda* cells served as a positive control. **E** Electron micrographs of purified *L. tarentolae*-derived NoV VLPs (scale bar: 100 nm). **F** Size distribution of NoV VLPs measured by DLS

Noroviruses are non-enveloped viruses which virons are composed mainly of one major structural protein VP1. A synthetic gene sequence of full length NoV VP1 capsid protein (the GII.4 NoV 2012 pandemic variant Hu/GII.4/Sydney/NSW0514/2012/AU) was optimized for the *L. tarentolae* using protozoan adapted codon (GeneArt—Thermo Fisher Scientific). The expression of the NoV VP1 protein was performed in high-density cell cultures (> 10^8^ cells/ml) using a tetracycline inducible LEXSY expression system. The synthetic gene was cloned into the pLEXSY_I-blecherry3 vector, with and without secretory signal peptide provided on the vector. Addition of secretory signal peptide did not change the localization of the protein (data not shown), which was predominantly in the cytoplasm. Protein expression was analyzed by western blotting in reducing conditions which confirmed that the molecular weight of the VP1 monomers was approximately 60 kDa (Fig. 1A). There is a faint band about 40 kDa which may correspond to the cleaved/degraded VP1 protein. The confocal microscopy analysis (Immunofluorescent Assay, IFA) showed a high level of NoV capsid protein, predominantly located in the cytoplasm of the *L. tarentolae* cells (Fig. 1B). ELISA assays clearly indicate that the NoV capsid protein expressed in *L. tarentolae* was specifically recognized by NoV genogroup 2 antibodies and by conformational antibodies against NoV capsid suggesting that recombinant VP1 protein self-assemble into VLPs (Fig. 1C). Proper folding and conformation of the VP1 protein was further confirmed by receptor binding assay (Histo-Blood Group Antigens (HBGAs) binding assay). The ability of NoV capsid protein produced in *L. tarentolae* cells to bind HBGAs was assessed by ELISA assay using mucin coated plates (Fig. 1D). Finally, *L. tarentolae-*derived NoV VLPs were purified and visualized using TEM and DLS. The obtained NoV VLPs were about 40 nm, which corresponds to the size of the native NoV (Fig. 1E, F).

### NoV VLPs produced in *L. tarentolae* elicit antibody responses in vaccinated mice

To demonstrate the immunogenicity of NoV VP1-based VLPs produced in *L. tarentolae*, two groups of BALB/c mice were immunized subcutaneously on days: 0, 14, and 28 with fractions containing NoV VLPs or PBS. All mice were immunized in presence of a squalene-based oil-in-water nanoemulsion adjuvant (Addavax). Two weeks after the last vaccination, the blood was collected and obtained sera were pooled in each group for further analysis.

**Fig. 2:**
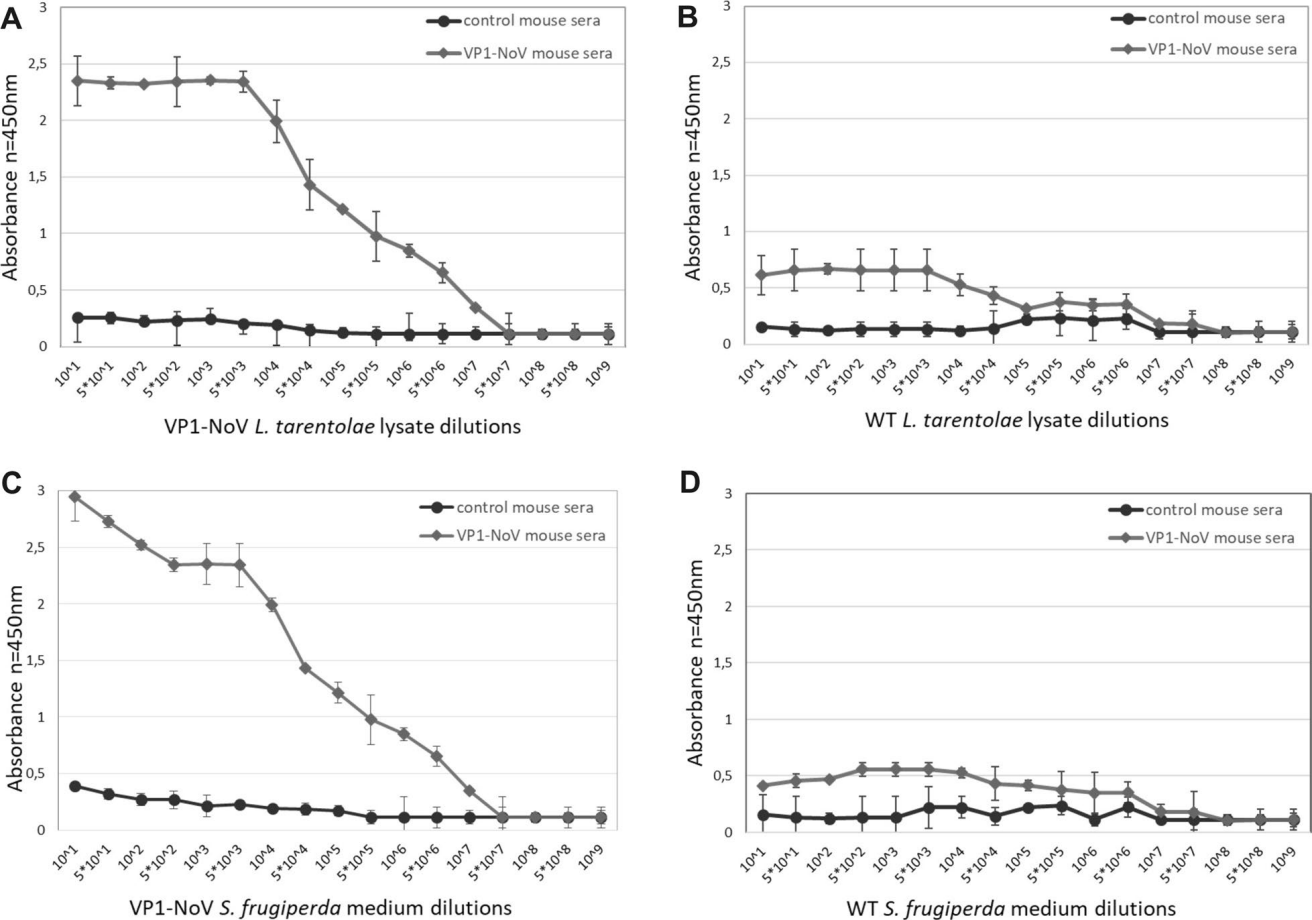
Analysis of the humoral response induced by NoV VP1-based VLPs in BALB/C mice. Recognition of VP1-NoV particles produced in *L. tarentolae* by pooled mouse sera collected after vaccination. The ELISA plates were coated with serial dilutions of recombinant *L. tarentolae* cell lysates containing VP1-NoV (**A**) or WT *L. tarentolae* cell lysates (**B**) (background threshold). As a reference *S. frugiperda* medium containing VP1-NoV (**C**) or WT *S. frugiperda* medium (D) was used. The dilution factor is depicted on x-axis. For each ELISA assay, the mean from three independent experiments performed is shown. The mean A450 values and standard deviations are shown on the y-axis

The humoral response induced by immunization was quantified by ELISA assay. Sera from mice immunized with PBS served as a negative control. The results confirm the ability to recognize the homologus and heterologous NoV recombinant protein VP1. High dilutions of NoV VP1 mouse sera were able to specifically recognize VP1 protein produced in different expression systems—protozoan *L. tarentolae* and insect *S. frugiperda* (WT cell lysates served as a background threshold) (Fig. 2).

**Fig. 3:**
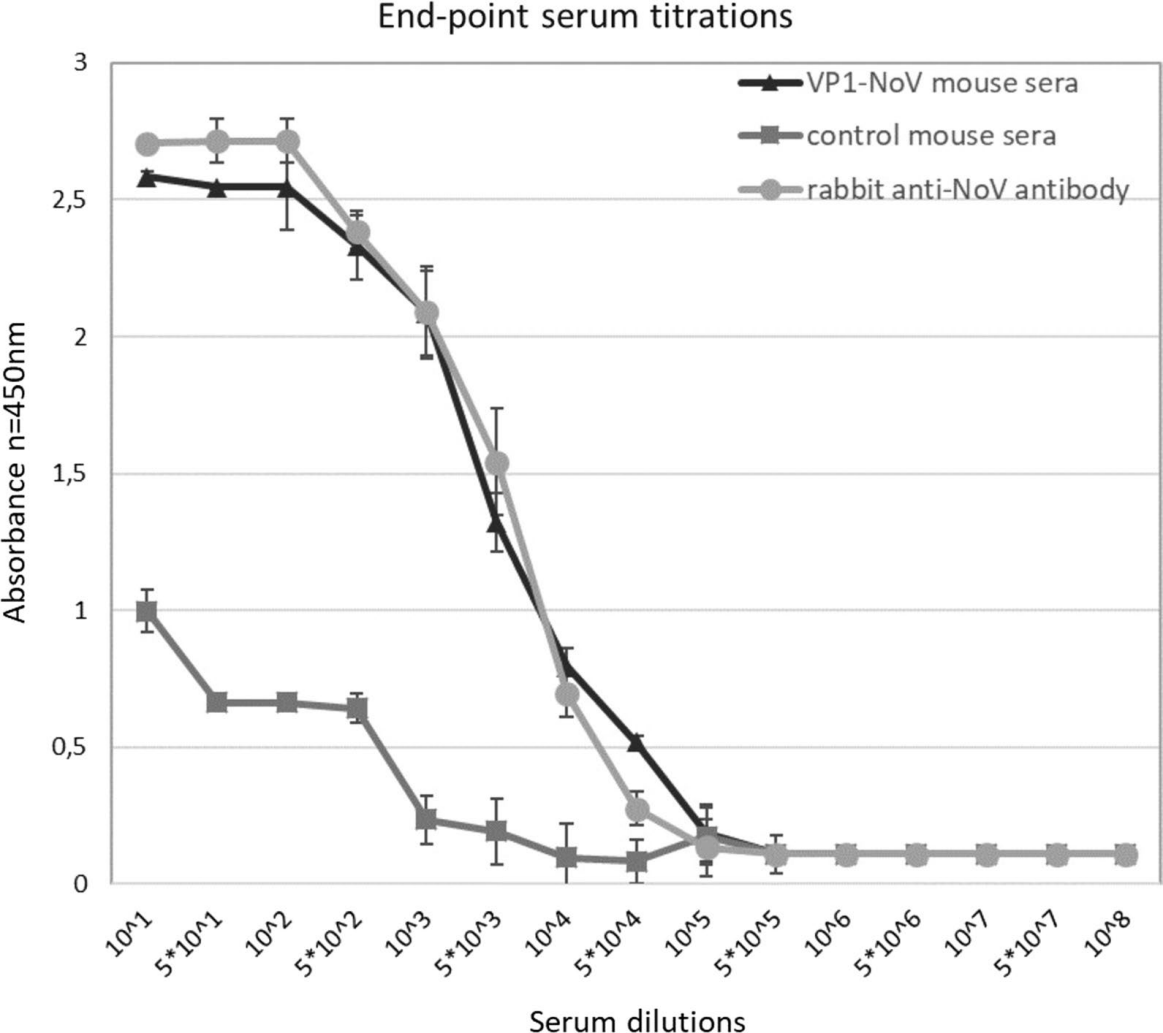
Analysis of the terminal antibody titers in the pooled mouse sera collected after immunization. ELISA plates were coated with *L. tarentolae* cell lysates containing VP1-NoV VLPs. The dilution factor of the pooled sera is shown on the x-axis. For each ELISA, the mean value from three independent experiments performed is presented. The mean A450 values and standard deviations are shown on the y-axis

The end point serum titrations show that the VP1-NoV antibody titer reached 10^4^ (Fig. 3). The antibody titer was estimated as the serum concentration at which the binding was at least 2 times higher than of the PBS-adjuvant immunized control serum.

**Fig. 4:**
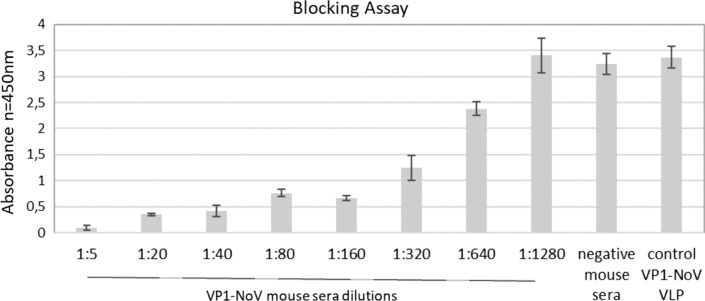
Analysis of VP1-NoV mouse sera in blocking assay. ELISA plates were coated with type III mucin from the porcine stomach. The ability of serial dilutions of mouse sera to block *S. frugiperda*-derived NoV VLPs to HBGAs was measured. The dilution factor of the pooled sera is shown on the x-axis. For each ELISA, the mean value from three independent experiments performed is presented. The mean A450 values and standard deviations are shown on the y-axis

HBGA blocking assays were conducted to test the neutralizing potential of obtained mouse antibodies. Type III mucin from the porcine stomach was used as a positive carbohydrate control since it was previously reported to contain primarily A and H antigens. Obtained results show a significant reduction of NoV VLPs binding to HBGAs after incubation with the mouse sera (PBS-vaccinated mouse sera served as a negative control). Antibody titers able to reduce VLP binding by > 50% (BT_50_) were observed for 1:5–1:320 serum dilutions (Fig. 4) confirming the specific protective immune responses induced by vaccination with NoV VP1-based VLPs produced in *L. tarentolae*.

## Discussion

Human NoVs are the main cause of acute viral gastroenteritis in people across all age groups. In June 2016, the World Health Organization Product Development for Vaccines Advisory Committee identified NoV as the priority pathogen for vaccine development [18].

VLPs are empty particles, which are unable to replicate and are morphologically identical to the native virus. VLPs elicit both cellular and humoral immune responses [9, 19]. Taking into consideration the high level of immunogenic properties of VLPs compared to the properties of soluble protein, the VLP platform is recently being employed for many vaccine studies. The NoV capsid protein VP1 possesses conformational immunodominant antigenic sites. These epitopes are presented in multiple copies on the surface of VLPs [7, 20]. VLP immunogens with an optimal presentation of these epitopes could potentially yield improved cross-protective immunity to human NoVs [21]. Thus, NoV VLPs produced by self-assembly of expressed VP1 seem to be a suitable candidate for NoV vaccine development [22].

The use of NoV VLPs as a vaccine candidate has been studied extensively, including a phase II clinical trial (NCT03039790), which may determine success in vaccine development against NoV infections [23–25]. This report is the first to describe the possibility of attaining efficient expression of NoV VP1-based VLPs in *L. tarentolae* protozoan parasite. Here, *L. tarentolae*-derived VP1 was properly folded which was confirmed by indirect immunofluorescence and ELISA assay using rabbit anti-NoV polyclonal antibodies. A biophysical analysis of the VP1 protein by ultracentrifugation in OptiPrep gradients revealed that the capsid protein self-assembles into the VLPs of approximately 40 nm in diameter confirmed by TEM (data not shown). These results are consistent with the previously published data about NoV VLP expression in yeast and insect cells [26, 27]. VLPs were mostly localized in the 33% OptiPrep layer which corresponds to the results described by Teixeira *et al.* were another member of the *Caliciviridae* family—the Rabbit Haemorrhagic Disease Virus (RHDV) was analysed by the same method [28]. The prime advantage of the LEXSY expression system is the possibility of low cost scaling up the cell culture and using bioreactors. These two features make *L. tarentolae*-based expression system attractive for industrial-scale antigen production [18]. Previously, this expression system was used for the production of only a small number of viral antigens, e.g., hemagglutinin, HPV L1 or small surface antigen of HBV [29–32], antibodies [17, 33–35] and recombinant proteins [36, 37].

The NoV VLPs produced in insect, mammalian, yeast [38], and *Escherichia coli* cells either as the full length VP1 protein [39, 40] or P particles [41, 42] have been previously reported to induce an antibody response in mice. Here, we present strong evidence of the immunogenic potential of VLPs produced in *L. tarentolae*. Sera of the mice immunized with parasite-derived NoV VLPs were able to specifically recognize the homologous-derived antigens as well as heterologous one from insect cells. Moreover, the level of the response was comparable for both types of VLPs. This cross-reactivity may suggest the similarity in the morphological structure. Analysis of the terminal antibody titers in obtained mouse sera revealed a high humoral response to the *L. tarentolae*-derived immunogen (titer 10^4^) which was comparable to the results for serum obtained from a rabbit immunized with VLPs produced in insect cells.

The goal of each vaccine is to induce the production of neutralizing antibodies that are able to block the entry of the virus. For NoV to enter HBGA receptors are utilized. To asses, if the *L. tarentolae*-derived VLPs are able to elicit protective response blocking assay was performed. We determined that mouse serum was able to strongly interfere with the ability of VLPs to interact with HBGAs. This data suggests that new parasite expression system can be successfully used for vaccine development.

## Conclusion

Presented results are the first to our knowledge to demonstrate that the *L. tarentolae*-derived VLPs formed by full-length NoV VP1 protein can induce a strong immune response and lead to the production of high titers of neutralizing antibodies. Moreover, successful expression of the recombinant particles in *L. tarentolae* could be of great importance in the search for an alternative solution serving a large-scale VLP production for pharmaceutical purposes.

## Materials and methods

### *Vp1* synthetic sequence and plasmid

The GII.4 NoV 2012 pandemic variant (Hu/GII.4/Sydney/NSW0514/2012/AU) *vp1* DNA coding sequence (1637 bp) was optimized using *L. tarentolae*-adapted codon (GeneArt—Thermo Fisher Scientific) and synthesized by Gene Art Gene Synthesis. Synthetized gene was ligated into BglII–NotI restriction sites in the *pLEXSY_I*-*blecherry3* vector (Jena Bioscience).

### *L. tarentolae* cultivation and expression

The NoV capsid protein was expressed using the inducible LEXSY expression system according to the manufacturer’s instructions (Jena Bioscience). Briefly, the plasmid carrying *Vp1* gene sequence was delivered into *L. tarentolae* cells by electroporation (450 V, 450 μF pulse time 5–6 ms). The electroporated cells were grown in suspension culture in LEXSY BHI medium supplemented with bleomycin (100 µg/ml). Subsequently, the recombinant cell line was cultivated in 25 cm^2^ tissue culture flasks filled with the selective medium at 26 °C and kept in the dark for 72 h, in agitated culture to the final optical density 4–5 at 600 nm (OD = 600). The T7 promoter-driven transcription was induced by adding tetracycline at the final concentration of 15 µg/ml.

### SDS-PAGE and western blotting

Samples containing NoV VLPs were loaded on 10–20% precast WedgeWell Gel (Thermo Scientific) and run at the constant voltage of 165 V. After electrophoresis, semi-dry electrotransfer of proteins onto polyvinylidene difluoride membranes was performed. Membranes were then blocked for 1 h in 5% semi-skimmed milk solution (5%milk/TBS/0.01% Tween20) and incubated overnight at 8 °C with rabbit anti-N terminal capsid protein of NoV antibodies (Abcam ab92976) (1:1000 in 5%milk/TBS/0.01%Tween20). The following day, the membranes were washed 3 times with washing buffer (TBS/0.01%Tween20) and incubated for 1 h at room temperature in a solution of Peroxidase-conjugated AffiniPure Goat Anti-Rabbit antibodies (Jackson Immuno Research) (1:4000 in 5%milk/TBS/0.01%Tween20). After washing (same as above) the reaction was developed with SuperSignal West Pico PLUS Chemiluminescent Substrate (Thermo Scientific).

### Immunofluorescence

*Leishmania tarentolae* cells expressing the NoV capsid protein and control cells were washed with PBS and fixed in 4% paraformaldehyde for 30 min at room temperature. Lysine-coated glass coverslips were covered with fixed cell suspension and left to dry. Next, the cells were permeabilized with 0.2% Triton X-100 in PBS for 10 min. Subsequently, the coverslips were incubated with rabbit anti-N terminal capsid protein of NoV antibodies (Abcam ab92976**)** (1:2000 in PBS/1%Tween20/5%FBS) for 1 h. The coverslips were then washed with PBS and incubated with Alexa Fluor 488-labelled goat anti-rabbit secondary antibodies (Invitrogen) (1:1000 in PBS/1%Tween20/5%BSA) for 1 h. After washing, the coverslips were mounted onto microscope slides with the ProLong Gold antifade reagent (Thermo Scientific) and the cells were analysed using a Leica TCS Sp8 X confocal microscope.

### VLP production and purification

#### Cell lysis

Tetracycline-induced *L. tarentolae* cell culture was grown in shake flasks for 72 h, at 26 °C, in agitated culture to the final optical density of 4–5 at OD600. Cells were then centrifuged for 15 min, 8000 rpm, at 4 °C. The cell pellet was resuspended in ice-cold lysis buffer (PBS/0.6%Tween-20) and sonicated (40 min, 40% amplitude, 10 s time on, 15 s time off). The lysed cells were centrifuged for 40 min, 8000 rpm, at 4 °C, the cell pellet was discarded, and the lysate was left for 16 h at 4 °C to allow particle formation.

#### Ultracentrifugation in a non-ionic iodixanol-based medium gradient (Opti-Prep™ Gradient)

The lysate containing VLPs was layered on OptiPrep™ gradient (Sigma-Aldrich) formed in ultra-clear tube (2 ml of 40% (v/v) OptiPrep™, 2.5 ml 36% (v/v) OptiPrep™, 2.5 ml 33% (v/v) OptiPrep™, 2 ml 30% (v/v) OptiPrep™ in ultra-clear water) and ultracentrifuged at 27,000 rpm for 16 h, at 4 °C. Then, 500 µl fractions were collected and analysed. The OptiPrep™ buffer was replaced with PBS using Amicon® Ultra 100 K centrifugal units (Merck Millipore). The purity of the fractions was evaluated by SDS-PAGE with Coomassie Brilliant Blue R-250 staining.

#### Detection of NoV VLPs using ELISA assay

A 96-well ELISA plate (Greiner Microlon High-Binding, clear) was coated with 100 µl/well of sequential dilutions of *L. tarentolae* cell lysates [wild type (WT) and NoV-VP1 lysates] and the coated plate was incubated overnight at 4 °C. Additionally, plate was coated with 100 µl/well of sequential dilutions of *S. frugiperda* medium [wild type (WT) and NoV-VP1] and incubated overnight at 4 °C. Then the plate was washed 4 × 5 min with 200 µl/well of washing buffer (PBS/0.05%Tween20), blocked for 1 h at 37 °C with 250 µl/well of blocking buffer (3%BSA/PBS/0.05%Tween20) and washed as previously. Next, 100 µl/well of mouse anti-NoV genogroup 2 antibodies (antibodies-online.com) (1:1000 in 3%BSA/PBS/0.05%Tween20), rabbit anti-VP1 conformational antibodies (Immune Technology Corp.) (1:1000 in 3%BSA/PBS/0.05%Tween20) or pooled sera from immunized mice collected on day 56 after immunization was added and incubated for 1 h, at 37 °C, and the plate washed as previously. Next, 100 µl/well of an Peroxidase-conjugated AffiniPure Goat Anti-Rabbit or Anti-Mouse antibodies (Jackson Immuno Research) (1:2000 in 3%BSA/PBS/0.05%Tween20) were added and incubated for 1 h at room temperature. Finally, following the last plate-washing step (6 × 5 min with 200 µl/well) 100 µl/well of HRP-substrate solution was added (1-Step Turbo TMB-ELISA, Thermo Scientific). The plate was incubated in dark until the blue color developed and the reaction was stopped by adding 50 µl of 0.5 M sulfuric acid to each well. Signal intensity was measured at 450 nm using a plate reader (NanoQuant Microplate Reader, TECAN).

#### Histo-blood group antigens (HBGAs) binding ELISA

A 96-well ELISA plate (Greiner Microlon High-Binding, clear) was coated with type III mucin from porcine stomach (Sigma-Aldrich; 10 µg/ml in PBS) for 4 h at room temperature. Next, the plate was washed 4 × 5 min with 200 µl/well of washing buffer (PBS/0.05%Tween20) and blocked for 2 h with 250 µl/well of blocking buffer (3%BSA/PBS/0.05%Tween20) at 37 °C. After blocking the plate was washed as previously. Next, 100 µl/well of NoV VLPs produced in *L. tarentolae* cells were added to the wells and incubated for 1 h at room temperature. The NoV VLPs produced in *S. frugiperda* cells served as a positive control. After washing, 100 µl/well of rabbit anti-VP1 conformational antibodies (Immune Technology Corp.) (1:1000 in 3%BSA/PBS/0.05%Tween20) were added and the plate was incubated for 1 h at 37 °C followed by incubation with 100 µl/well of a Peroxidase-conjugated AffiniPure Goat Anti-Rabbit antibodies (Jackson Immuno Research) (1:2000 in 3% BSA/PBS/0.05%Tween20) for 1 h at room temperature. Finally, the plate was washed (6 × 5 min with 200 µl/well) and 100 µl/well of HRP-substrate solution was added (1-Step Turbo TMB-ELISA, Thermo Scientific). The plate was incubated in dark until the blue color developed, and the reaction was stopped by adding 50 µl of 0.5 M sulfuric acid to each well. Signal intensity was measured at 450 nm using a plate reader (NanoQuant Microplate Reader, TECAN)**.**

### Electron microscopy

In order to visualize the VLPs produced in the *L. tarentolae* partially purified lysates were diluted in the TM buffer (50 mM Tris–HCl pH = 7.4/10 mM MgCl_2_) and adsorbed onto carbon-coated grids. Negative staining was performed using 2% uranyl acetate. The particles were examined in a Philips CM100 transmission electron microscope (TEM; University of Gdańsk, Poland).

### Dynamic light scattering (DLS)

Particle sizing was performed using a Malvern Instrument Zeta Sizer NanoS DLS instrument (Malvern, Worcestershire, UK). Measurements were taken in water at 25 °C. Sample solutions were passed through 0.45 μm filters and equilibrated at room temperature for 10 min prior to measuring, where the duration of each measurement was 10 s. The results were calculated as the average of six consecutive measurements.

### Animal immunization

Two groups of 6 BALB/c male mice (6 weeks old) were immunized subcutaneously. Frist group were mice serving as negative controls and were immunized with a 1:1 PBS-adjuvant mixture at time point 0, 14, 28 days. Second group was immunized*.* with 15 µg of VLPs produced in the *L. tarentolae* at day 0 and with 10 µg of capsid protein at 14 and 28 day Potential vaccine candidate was mixed in the 1:1 ratio with squalene-based oil-in-water nanoemulsion adjuvant (Addavax, InvivoGen). All experiments on animals were conducted by an accredited company (Tri-City Central Animal Laboratory Research and Service Center, Medical University of Gdańsk) in accordance with the current guidelines for animal experimentation. The protocols were approved by the Committee on the Ethics of Animal Experiments of the Medical University of Gdańsk (Permit Number: 45/2015). All surgery procedures were performed under isoflurane anaesthesia, and all efforts were taken to minimize animal suffering.

#### End point titration of mouse sera by ELISA assay

Sera from the immunized mice were collected and pooled on day 56 after immunization. A 96-well ELISA plate (Greiner Microlon High-Binding, clear) was coated with 100 µl/well of  *L. tarentolae-derived NoV- VP1 *cell lysates. The coated plate was incubated overnight at 4 °C. Next, the plates were washed 4 × 5 min with 200 µl/well of washing buffer (PBS/0.05%Tween20) and blocked for 2 h with 250 µl/well of blocking buffer (3%BSA/PBS/0.05%Tween20) at 37 °C. The plates were washed as previously and serial dilutions of mouse sera (in 3%BSA/PBS/0.05%Tween20) were added to the wells and incubated for 1 h at room temperature. Serial dilutions of rabbit anti-NoV antibodies (Abcam ab92976**) (**in 3%BSA/PBS/0.05%Tween20) served as a positive control. After incubation plates were washed as previously, and appropriate secondary antibody solution (Jackson Immuno Research) (in 3%BSA/PBS/0.05%Tween20) was used for detection. Finally, following the last plate-washing step (6 × 5 min with 200 µl/well), 100 µl/well of HRP-substrate solution was added (1-Step Turbo TMB-ELISA, Thermo Scientific), the plate was incubated in dark until the blue color developed, and the reaction was stopped by adding 50 µl of 0.5 M sulfuric acid to each well. Signal intensity at 450 nm was measured using a plate reader (NanoQuant Microplate Reader, TECAN)**.**

#### Blocking ELISA

A 96-well ELISA plate (Greiner Microlon High-Binding, clear) was coated with type III mucin from porcine stomach (Sigma-Aldrich; 10 µg/ml in PBS) for 4 h at room temperature. Next, the plate was washed 4 × 5 min with 200 µl/well of washing buffer (PBS/0.05%Tween20) and blocked for 2 h with 250 µl/well of blocking buffer (3%BSA/PBS/0.05%Tween20) at 37 °C. Then, serially diluted mouse sera (starting dilution, 1:5) were mixed with an equal volume of NoV VLPs produced in *S. frugiperda* insect cells and incubated for 1 h at room temperature. As a negative control the serum collected from PBS-vaccinated mice was used. Afterward, the VLP-serum mixture was added to the mucin-coated plate and incubated for 1 h at room temperature. The NoV VLPs produced in *S. frugiperda* without addition of mouse sera served as a positive control. After washing, 100 µl/well of rabbit anti-VP1 conformational antibodies (Immune Technology Corp.) (1:1000 in 3%BSA/PBS/0.05%Tween20) were added and incubated for 1 h at 37 °C followed by incubation with 100 µl/well of an Peroxidase-conjugated AffiniPure Goat Anti-Rabbit antibodies (Jackson Immuno Research) (1:2000 in 3%BSA/PBS/0.05%Tween20) for 1 h at room temperature. Finally, the plate was washed (6 × 5 min with 200 µl/well) and 100 µl/well of HRP-substrate solution was added (1-Step Turbo TMB-ELISA, Thermo Scientific). The plate was incubated in dark until the blue color developed, and the reaction was stopped by adding 50 µl of 0.5 M sulfuric acid to each well. Signal intensity was measured at 450 nm using a plate reader (NanoQuant Microplate Reader, TECAN)․ The BT50 was defined as the serum dilution at which the OD450 value was 50% of that of the positive control (VLPs only).

### NoV positive control

Purified NoV VLPs were produced in *S. frugiperda* cells as described before [43]. Briefly, synthetic DNA coding *Vp1 c*apsid protein of the GII.4 NoV 2012 variant (Hu/GII.4/Sydney/NSW0514/2012/AU) was synthesized by Gene Art Gene Synthesis (Thermo Fisher Scientific) and cloned into the baculovirus transfer vector pFastBac1 (Invitrogen, Carlsbad, CA) to obtain recombinant baculovirus (rBV-VP1) in *Sf9* insect cells. For the production of NoV VLPs, *Sf9* cells in suspension culture were infected with rBV-VP1 at an MOI of 3 and harvested 60 h post-infection.

### Statistical analysis

All Statistical analyses were performed using Sigmaplot 12.0 software (SYSTAT Software). Statistical differences between the means of the two groups were analyzed using a t-test. Each experiment was performed in triplicates and the P value < 0.05 was considered to be statistically significant.

## Data Availability

The datasets used and/or analysed during the current study are available from the corresponding author on reasonable request.
